# General Designs Reveal Distinct Codes in Protein-Coding and Non-Coding Human DNA

**DOI:** 10.3390/genes13111970

**Published:** 2022-10-28

**Authors:** Dana Cohen

**Affiliations:** Ronin Institute, 127 Haddon Pl, Montclair, NJ 07043-2314, USA; dana.cohen@ronininstitute.org

**Keywords:** general designs, genomic signature, dinucleotide, DNA codes, protein coding, non-coding, DNA sequence, purine pyrimidine, weak strong

## Abstract

This study seeks to investigate distinct signatures and codes within different genomic sequence locations of the human genome. The promoter and other non-coding regions contain sites for the binding of biological particles, for processes such as transcription regulation. The specific rules and sequence codes that govern this remain poorly understood. To derive these (codes), the general designs of sequence are investigated. Genomic signatures are a powerful tool for assessing the general designs of sequence, and cross-comparing different genomic regions for their distinct sequence properties. Through these genomic signatures, the relative non-random properties of sequences are also assessed. Furthermore, a binary components analysis is carried out making use of information theory ideas, to study the RY (purine/pyrimidine), WS (weak/strong) and KM (keto/amino) signatures in the sequences. From this comparison, it is possible to identify the relative importance of these properties within the various protein-coding and non-coding genomic locations. The results show that coding DNA has a strongly non-random WS signature, which reflects the genetic code, and the hydrogen-bond base pairing of codon–anti-codon interactions. In contrast, non-coding locations, such as the promoter, contain a distinct genomic signature. A prominent feature throughout non-coding DNA is a highly non-random RY signature, which is very different in nature to coding DNA, and suggests a structural-based RY code. This marks progress towards deciphering the unknown code(s) in non-protein-coding DNA, and a further understanding of the coding DNA. Additionally, it unravels how DNA carries information. These findings have implications for the most fundamental principles of biology, including knowledge of gene regulation, development and disease.

## 1. Introduction

### 1.1. Protein-Coding Verses Non-Coding DNA, Distinct Functionality Leads to Different Codes 

Protein-coding regions of the genome comprise less than 2% of the genomic DNA sequence in the human. This is a small, yet, critically important component of the genome. These sequences are well-understood. These function via the famous triplet code, whereby the information content is three bases specifying the translation of a sequence into amino acids, which make up proteins. 

Non-coding DNA comprises a huge amount of a sequence, and these have a very a different function to protein-coding sequences. Within the non-coding category of sequences, there are also distinct regions with separate functionalities. However, in a general sense, these are responsible for gene regulation. The encoding for these sequences is very different to protein-coding sequences, and remains elusive [1].

Within the vast landscape of non-coding sequences are contained many different elements responsible for gene regulation. As part of the transcription machinery, upstream of the coding sequence human genes lies the promoter, which initiates transcription via PolII, and possesses multiple transcription factor binding sites (TFBS’s) [2,3]. Non-coding DNA is also populated with a multitude of enhancer elements which regulate transcription in a tissue-dependent manner [4,5], and these are also dense in potential TFBS’s. These are typically dense around the promoter, but may also occur at great distance from it. In addition, there are non-coding RNAs which form RNA particles which have potential roles in transcription, post-transcription regulation and epigenetics, and more [6,7]. Therefore, a high proportion of non-coding DNA contains sequences associated with gene regulation. 

The basis of the non-coding DNA code(s) are mostly (but not exclusively) related to protein binding to DNA, and this recognition. In contrast, the basis of protein-coding DNA codes is the rule-driven triplet code. Since protein-coding and non-coding DNA have very different functions, they possess distinct patterns and codes within their sequences [8,9]; therefore, the characteristics of the sequence are also expected to be distinct [10]. 

Experiments including very early studies assessed the various frequency measurements of triplets and frequencies such as RNY > RNR > YNY > YNR were found to be general occurrences within coding sequences [11,12], as well as the phenomenon of the third base wobble. These relate to the triplet code, and may be due, for instance, to use of some codons over others. Frequency measurements alone, whilst important, are in other ways limited in what they tell us about sequence patterns and unlikely codes. 

### 1.2. Genome Architecture and General Designs

There exists an inherent connection between sequence, structure and function [13,14]; therefore, analysis of DNA sequences and their signatures permits greater understanding of structure and function. Genomic signatures are a powerful tool for characterizing DNA [15]. This can distinguish non-coding regulatory DNA, as well as identifying the differences to coding DNA [16,17]. Furthermore, patterns and signatures help build rules for how these sequences are constructed/assembled. This marks a step forward towards deciphering more specific regulatory codes, as it provides a set of sequence characteristic “rules”, which can be built upon in a step-wise manner. 

Long-range correlations (using DNA-walk) are known to exist in DNA [18] in many organisms, and these also have a fractal-like nature [19]. This is within intron-containing genes, and is the case regardless of the type of protein coding sequence, and also within non-transcribed regulatory regions. Such correlations include features such as GC-rich isochores [20].

Genomic signatures (Dinucleotide relative abundance profiles) are pervasive and stable in genomes [21]. It is thought that the reason for this may be the existence of genome-wide factors. Examples include the replication and repair machinery, mutational tendencies and structural tendencies of genomic DNA. Dinucleotides are the most basic unit/description of a sequence, and can also be utilized to determine non-random properties of sequences. This is because dinucleotides may be suppressed (under-represented) or enhanced (over-represented) [22,23]. Dinucleotide relative abundance profiles show a departure from the randomness of genomic DNA sequences and collectively form a distinct genomic signature [24]. Early research [25] has shown nearest neighbour (dinucleotide) effects in prokaryotic and eukaryotic genomes. Here, it was observed that certain general features occur in genomes. CpG and TpA are suppressed, and also with this an asymmetry exists in the sequences. For instance ApT > TpA, CpT > TpC, TpG > GpT and GpC > CpG occur in examined genomes, including both prokaryotes and eukaryotes. Chargaff’s second parity rule states that: A~T and C~G in single stranded DNA [26], and, despite this, in the dinucleotides we see suppression/enhancement.

Both coding and non-coding DNA sequences possess an inherent non-randomness [27]. Non-randomness may also be connected with sequence functionality. This is because sequence function, by its nature, enlists non-random patterns or codes. It utilises a language of DNA, so to speak. Compositional differences have also been analysed in this way within organisms, such as the comparison between mitochondrial and nuclear genomes. Dinucleotides and their relative abundance profiles can, therefore, be used genome-wide as a powerful tool to analyse the general sequence designs and differences between non-coding and coding DNA. This may also be further extended to different types of non-coding DNA.

### 1.3. An in-Depth Look at DNA-Nucleic Acids: Beginning to Decipher Codes through Components

Information is stored within nucleic acids in the form of four different chemical bases [28]. The well-known triplet code within coding sequences utilises these four chemical bases in various combinations with some redundancy to generate specific amino-acid sequences for peptides. This is performed via the tRNA. The same four-base information content ‘alphabet’ is utilized, albeit differently in regulatory DNA sequences. However, in order to understand a different “coding” system, it is helpful to break down the information content, and simplify it as much as possible into its components. 

**Figure 1:**
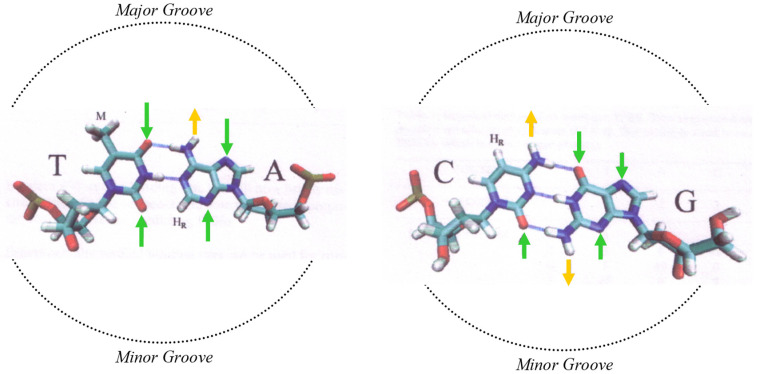
Diagram of base pairs of DNA, and recognition patterns for hydrogen-bond donor and acceptor sites. This is adapted from a diagram by Höglund et al., 2004 [30] (see Appendix A for article URL and licensing). These patterns are shown for the major and minor grooves of B-DNA. The four DNA bases, A, T, C and G, each contain distinctive and yet overlapping chemical and physical properties. There are three separate categories of property: The first is the weak or strong hydrogen-bonding capacity of the DNA bases. This is with reference to the number of hydrogen bonds between the base pairs of the DNA. A and G are weak (W) bases because these contain only two hydrogen bonds between the complementary base pairs, and C and G are strong. The second category is the purine or pyridine structural property. The DNA bases contain either a two-ring or one-ring molecular structure. The two-ring structures are called purines, and A and G are purines (R). The one-ring structures are pyrimidines; C and T are pyrimidines (Y). The third property is the chemical property of an amino or keto group. The bases each contain one of these. The bases A and C contain an amino (M) group, whereas G and T contain a keto (K) group. This affects the hydrogen-bonding capability of the bases, and, specifically, the hydrogen-bonding donor/acceptor patterns positioned at the major groove of the DNA. Hydrogen-bond acceptors are shown by green arrows and donors are shown by yellow arrows. In the minor groove, it is only possible to distinguish a C-G base pair from a T-A base pair via these hydrogen-bonding patterns. In the major groove, it appears possible to distinguish all four bases.

The chemical bases, and their information content, can be sub-divided into a binary system, much like the way in which a computer stores binary information, as described by information theory [29]. This is extremely useful as a way to decipher underlying codes, as it breaks down complex information into a simpler form, or components. When doing so with DNA sequences, this sub-division of information is based on the real chemical or physical properties of the nucleic acids and base pairs. There are three well-defined types of property of the bases [30,31], and these are used in our research as a means to break down the information content of DNA (see Figure 1).

A conversion of DNA sequences from an ATCG sequence into each of the RY, WS, and KM sequences represents a breakdown of information content, which isolates one property of the bases in the process. It, therefore, binarized the sequences. This, then, permits an analysis of each of these properties of DNA separately. It has the advantage of allowing us to see patterns and signatures (sequence designs) in isolation.

One type of DNA property is the purine/pyrimidine chemical property. This describes the chemical-ring structure of the bases, with purines possessing a two-ring structure, and pyrimidines one ring. The purine/pyrimidine content of DNA is known to influence the secondary structure of DNA [32].

Binarizing the DNA sequence for this chemical property means converting the four (ATCG) bases into purines and pyrimidines (RY). A and G are purines (R), and C A and G are pyrimidines (Y). The second binary conversion is into weak or strong bases (WS), which define hydrogen bonds between the base pairs. C and G are strong (S) bases, as there are three hydrogen bonds between the base pairs, and A and T are weak (W), as there are two hydrogen bonds between base pairs. This property also affects potential hydrogen-bond donor/acceptor sites in the minor groove of DNA. The third is keto and amino bases (KM), which reflects tautomerism, and the hydrogen-bond donor–acceptor patterns in the major groove of DNA. The bases A and C contain an amino (M) group, whereas G and T contain a keto (K) group. In general, RY is a physical structural property whilst WS/KM is a chemical property due to hydrogen-bonding potential.

In our previous research [33], we developed a method to analyse DNA using genomic signatures in conjunction with binarized sequences. This method studies different sequences’ chemical and physical properties for relative non-random features. We called this a components analysis, where general designs are applied to RY, WS, and KM binarized sequences. This provided a powerful method for extracting patterns and codes from the sequences. This was a large-scale genomic sequence analysis. The results revealed a general and pervasive RY structural code within human chromosomal DNA. This was strongly present in all chromosomes. Here, we extend the same components analysis and apply it to separate categories of coding and non-coding DNA, and a variety of distinct genomic sequence types. This is performed in order to understand the general encoding of these distinct regions of the genome, and observe how these differ from each other.

### 1.4. Aim of Experiment and Importance of Findings

The aim of this research is to characterize DNA signatures within distinct coding and non-coding regions of the human genome. These include coding DNA, transcript, the promoter, enhancer, 5′UTR and 3′UTR. This permits progress towards deciphering DNA codes. This is carried out via a large-scale sequence analysis, which permits general and pervasive signatures to be observed.

The DNA sequences are converted into their binary components RY/WS/KM, as described above, so that individual DNA/base properties could be isolated. We refer to this as a binary components analysis. Dinucleotide relative abundance profiles are calculated, and the non-randomness of sequences observed. This enables cross-comparisons of general designs to be made between the three binary components. In addition, the distinct functional genomic locations, such as coding DNA and UTRs, are cross compared. Genomic signatures are analysed, and biological meaning is derived from sequence data, providing a better understanding of the patterns and codes underlying genomic locations of different functionality.

The findings of this research reveal that the promoter contains a distinct genomic signature, which is different to other non-coding DNA, and all non-coding regions are very distinct from coding DNA. Furthermore, the promoter region contains a highly non-random RY signature, which is very different in nature to coding DNA, and points towards an RY code. This result has implications for transcription regulation, and suggests that non-coding DNA, in general, has an RY code which is more pronounced in the promoter. The RY sequence is a strong determinant of structure, including DNA secondary structure, rigidity/flexibility, and ability to bend. Therefore, this result points towards a structural-based RY code in the promoter/intergenic region. The protein-coding DNA is very different in general designs and binary components analysis to non-coding DNA. Here, we observe a general WS code, which connects to the hydrogen-bonding property of the bases. This reflects the nature of the triplet code, which functions primarily via the complementary base pairing of the tRNA. 

The binary components analysis captures the inherent codes and properties of the DNA, strongly differentiating between coding and non-coding DNA. This is a novel investigation, utilising general designs with a binary components analysis to study distinct sequence types, and the results obtained are novel and important. Transcription regulation lies at the central dogma of biology, and is key to understanding basic cell function, development and disease. Understanding the different encodings of distinct genomic functional regions is crucial to biology, and how the genome functions. 

## 2. Methods and Concepts 

### 2.1. Obtaining Genomic Sequence Datasets

DNA sequence datasets using for this analyses were obtained from the Ensembl human genome build 38 database (genome assembly GRCh38.p5). The goal was to obtain different functional sequence types. The sequences obtained from this source include coding, transcript, 5′UTR and 3′UTR, and these datasets were extracted utilising BioMart queries [34]. Through these queries, coding, transcript, 5′UTR, and 3′UTR sequences were obtained. 

For the BioMart queries, the parameters were set to ‘GENES’ and the filters were Gene type: protein_coding and Status (gene): KNOWN. This limited the results to protein-coding genes only and to known genes. This resulted in a dataset of 22,078/66,232 genes. This filter served to limit the output, and increase accuracy of downstream analysis. With these parameters, protein-coding and full transcript sequences were extracted as well as the 5′UTR and 3′UTR. The transcript sequences were full transcripts including intronic sequences.

The enhancers and promoters were taken from different sources in order to obtain datasets of optimal accuracy. The enhancers were obtained from the Vista database [35]. This contains a dataset of experimentally verified human enhancers. Whilst it is a minimal dataset, utilizing this minimizes prediction and increases accuracy of the sequence set used. A total of 1942 elements were downloaded from the VISTA enhancer browser. For the promoters, the Eukaryotic Promoter Database (EPD) was used and human elements extracted from this [36,37]. A total of 16,454 human promoters were obtained and utilised from this source. This database has the advantage that it is a non-redundant set of eukaryotic POL II promoters, with experimental verification for the TSS (transcription start site). This verification greatly increases accuracy, and assists in removing error and noise from the downstream analysis. 

For each of the sequence datasets, coding, transcript, 5′UTR and 3′UTR, enhancer and promoter, and equivalent random model was generated. This random model contains the equivalent number of sequences and each one is of the same sequence length, in each of the individual datasets. For each real sequence, a randomised or shuffled sequence is generated, and this sequence contains the same frequency of bases; however, these are shuffled into a randomised order. To achieve this, the EMBOSS ‘Shuffleseq’ tool was used [38,39]. Within each sequence-type dataset, each individual sequence was randomised with Shuffleseq to generate a random sequence, and the process was repeated for all the sequences in the dataset. This then permitted the sequence analysis to be carried out with a randomised set, as well as a real sequence set. 

### 2.2. Sequence Processing

For each coding, transcript, 5′UTR, 3′UTR, promoter and enhancer sequence datasets, a Python script was written and employed to process the DNA sequences. This was performed for calculating mononucleotide and dinucleotide frequency, as well as odds ratios, relative abundance and distance from randomness values. For each genomic location, the individual sequences were taken as separated entities for the calculations. The dinucleotide frequencies, odds ratios, and relative abundance calculation, and distance from randomness were taken for every sequence in a given dataset.

Sequence processing was carried out for the coding, transcript, 5′UTR, 3′UTR, promoter and enhancer datasets separately. Within each of these six datasets, dinucleotide and also mononucleotide frequencies were worked out for each of the individual sequences. Mononucleotides frequencies are a count of the classic A, T, C, G chemical bases represented by these four letters. The dinucleotide formed by these are a total of 16 possible combinations to include; ApA, ApT, ApC, ApG, TpA, TpT, TpC, TpG, CpA, CpT, CpC, CpG, GpA, GpT, GpC and GpG, (see Supplementary Materials for definitions). Each of these dinucleotides was determined stepwise along the sequence from the 5′ to the 3′ along the transcribed strand. The total number of dinucleotides (and mononucleotides) depends on the sequence length, which varies in the datasets. 

Descriptive statistics were initially calculated for each of the six genomic-region datasets. This included the mean values, median, standard deviation, variance, and quartile, and range for all the fragments within each genomic sequence type. This resulted in statistics and analysis for each location separately.

### 2.3. Genomic Signatures 

The dinucleotide representation in a sequence can be used to assess dinucleotide contrasts whilst taking into consideration the mononucleotide composition of the sequence. This describes the proportion of each dinucleotide, above or below the random expectation. This representation value is calculated using an odds ratio. The odds ratio can also be referred to as a single-strand dinucleotide relative abundance ratio.

Dinucleotide odds ratio: ρ_XY_ = f_XY_/f_X_f_Y_.

For any given nucleotide X, fx is the frequency of the X, within the sequence. fxy is the frequency of the dinucleotide XpY within that sequence. An odds ratio value of ρxy >> 1 indicates enhancement or over-representation of the dinucleotides beyond the random expectation. In contrast, an odds ratio value of ρxy << 1 indicates suppression or under-representation of that dinucleotide. In a random sequence (i.e., a shuffled sequence), the ρxy values for all the dinucleotides approach 1.0. The longer the random/shuffled sequence, the closer the odds ratio value would approach the theoretical random expectation of 1. 

The odds ratios of the 16 dinucleotides form dinucleotide relative abundance profiles, whose difference from 1 provide a measure of deviation from randomness. Collectively, the odds ratios are said to be the general designs of given sequences. The genomic signature extends the use of general designs to measure the difference between two distinct sequence types. This is called average absolute dinucleotide relative abundance difference, and it is worked out as follows: 

Average absolute dinucleotide relative abundance difference:

$$\delta \;(f,\;g)\;=\;1/16\sum_{\mathrm{XY}}|\rho \mathrm{XY}\;(f)\;-\;\rho \mathrm{XY}\;(g)|$$

Here, f is one sequence type whereas g is another. The sum here extends over all sixteen possible dinucleotides. o is used to demonstrate a departure from randomness of genomic DNA sequences. In this experiment, the average absolute dinucleotide relative abundance is further adapted to measure the difference between a real genomic sequence and a theoretical randomized one. This permits a measure of distance from randomness, or comparison of sequence to a random model. 

Distance from randomness:

$$\lambda \;(f,\;g)\;=\;1/16\sum_{\mathrm{XY}}|\rho \mathrm{XY}\;(f)\;-\;1|$$

Here, the sum extends over all dinucleotides. Therefore, this calculation is equivalent to the mean of dinucleotide odds ratio values for all 16 possible dinucleotides for a given sequence. We refer to this calculation as the ‘distance from randomness’. The further this value is from 0, the further away the value is from the random (or equivalent shuffled) sequence. This total distance from randomness is calculated for each individual sequence, within each of the six datasets (coding, transcript, 5′UTR, 3′UTR, promoter and enhancer) examined. 

### 2.4. Binary Components Analysis

In our previous paper, we discuss the concept of binary components analysis in greater depth [33]. Here, we summarise the methods used: In order to analyse these (purine/pyrimidine verses weak/strong, and keto/amino) nucleotide properties separately, the ATCG upstream sequence was translated into two different but equivalent sequences. Therefore, a dataset is generated for each of the three binary components, RY, KM and WS; then, the original ATCG native DNA sequence is converted or binarized, according to these properties. This is performed for each of the six different types of genomic sequence studied. Therefore, the sequence datasets are ‘translated’ into three separate sets for RY, KM and WS. The first is the translation of the original ATCG sequence to a purine/pyrimidine (R/Y) sequence: A and G are converted to purines and C and T are converted to pyrimidines (R). For the second set, the ATCG sequence is converted to a weak/strong (W/S) sequence, where C and G are converted to strong (S) bases, and A and T are converted to weak (W) bases. For the third and final conversion, the native sequence is converted to a keto/amino (K/M) sequence. Here, the bases A and C are converted to an amino (M)-type base, whereas G and T contain a keto (K) group base. 

These conversions, or ‘translations’, result in three binarized sequences for each of the original native ones. These are then treated as separate entities for analyses. This is performed so that the relative importance of these three subdivisions of nucleotide properties could be assessed individually. For each of these three binary components datasets, RY, KM and WS, there are four possible dinucleotides (instead of the sixteen). The dinucleotides for each of these datasets are as follows:

RY binary sequence dataset: RpR, RpY, YpR, YpY

WS binary sequence dataset: WpW, WpS, SpW, SpS

KM binary sequence dataset: KpK, KpM, MpK, MpM

For each of the RY. WS, and KM binary-components (three-sequence) types, distance from randomness values are calculated taking into consideration its specific mononucleotide and dinucleotide composition. Distance from randomness results are then averaged (mean values) over the entire dataset of sequences. This is repeated for the codon, transcript, 5′UTR, 3′UTR, promoter and enhancer.

The genomic signature and distance from randomness are adapted for the binary components analysis, and the binary sequences. The dinucleotide frequencies, odds ratios and genomic signature calculations are carried out on the translated sequences, with the above-outlined 4 dinucleotides for each of the binarized sequences. This is instead of the 16 dinucleotides for the native DNA sequences. This time, though, the dinucleotides are changed accordingly. Therefore, the distance from randomness calculation is given below:

Average absolute dinucleotide relative abundance difference for the binary components would be:

$$\delta \;(f,\;g)\;=\;1/4\sum_{\mathrm{XY}}|\rho \mathrm{XY}\;(f)\;-\;\rho \mathrm{XY}\;(g)|$$

Therefore, the distance from randomness is:

$$\lambda \;(f,\;g)\;=\;1/4\sum_{\mathrm{XY}}|\rho \mathrm{XY}\;(f)\;-\;1|$$

The sum here extends over all four possible dinucleotides. 

### 2.5. Statistical Analysis: Significance Testing

Statistical testing was carried out to determine whether there is significant difference between the RY, KM and WS binary component sequence datasets. This significance testing was performed using IBM SPSS statistics software. The effects of the three different RY, WS, and KM binary components were compared in each of the different genomic sequence datasets. Therefore, the ANOVA’s were performed for each of the coding, transcript, 5′UTR and 3′UTR, enhancer and promoter datasets. Six separate one-way within-subject ANOVA’s were performed (at the 1% level of significance), comparing the distance from randomness (relative abundance profiles) for RY, WS and KM in each sequence dataset separately (see Supplementary Materials). The Maulchy test was applied to evaluate sphericity. Since the sphericity assumption was violated in the cases, the Greenhouse–Geisser correction was applied. The ANOVA’s were followed up with post-hoc multiple pairwise comparisons to evaluate and then explore the differences between binary components. Here, multiple pairwise comparisons were made and corrected by the Bonferroni method, since it does not assume independence and was most appropriate for these datasets. 

The hypothesis testing within each individual genomic sequence type is outlined as:

**H0:**  *There is no significant difference between the binary components RY, KM and WS profiles in a given sequence type.*

**H1:**  *There is a significant difference between the RY, KM and WS binary components profiles.*

In addition, the difference between the distance from randomness (dinucleotide relative abundance) profiles for the real DNA sequences verses the randomised-shuffled equivalent sequences was calculated. This was carried out for all six genomic sequence datasets individually, using paired *t*-tests (two-tailed at the 1% level of significance). 

The hypothesis testing for real verses random models is outlined as:

**H0:**  *The null hypothesis of is that there is no difference between the real-native, and randomized shuffled datasets.*

**H1:**  *The alternate hypothesis is that there is a significant difference between real and randomised sequences.*

## 3. Results 

### 3.1. Distance from Randomness of Different Genomic Locations: Original ATCG Sequence

**Figure 2:**
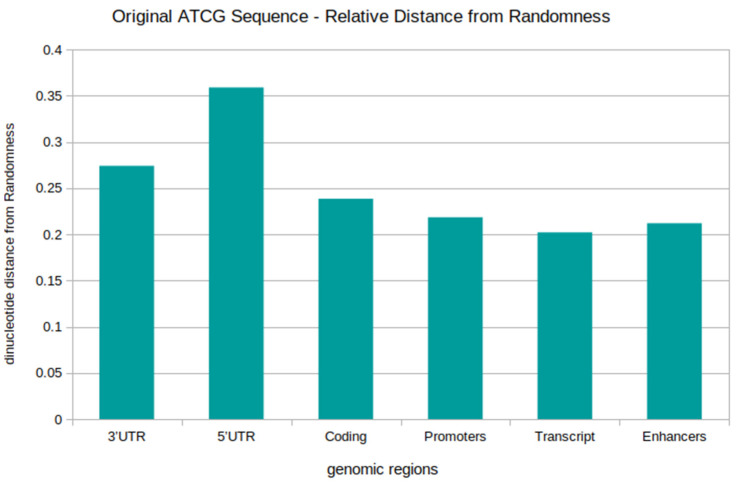
Distance from randomness of different genomic regions for the ATCG original sequence: The profile shows the average (mean) distance from randomness values of the different genomic DNA sequences, within a given region. The sequences analysed include the 3′UTR, 5′UTR, coding, promoter, transcript, and enhancer. A value of zero denotes the random model, and the further away the values from zero, the greater the distance from randomness. The different genomic regions each display a distinct distance from randomness value and profile; however, all are non-random in nature. This reflects that the difference in profiles reflects sequence properties and patterns of each region. The UTR’s are most distant from the random model with respect to all the possible dinucleotides, with the 5′UTR being the most non-random in nature, followed by the 3′UTR. This is then followed by coding sequences. Promoter and enhancers are relatively similar, and transcript is closest to ‘randomness’. Overall, this demonstrates that different genomic regions, with different functionalities, possess distinct sequence features, in a general sense, and this is likely due to functionality itself with its own encoding, as well as distinct sequence assembly and evolutionary constraints. The UTR’s are most distant from the random model with respect to all the possible dinucleotides, with the 5′UTR being the most non-random in nature, followed by the 3′UTR. This is then followed by coding sequences. Promoter and enhancers are relatively similar, and transcript is closes to ‘randomness’.

In order to identify unique signatures and the general non-random quality of the different sequence types, distance from randomness (dinucleotide relative abundance profiles) measures were taken for the original ATCG native sequences. This was analysed for the genomic locations of distinct functionality, namely; coding sequences, transcript, promoter, enhancer, 3′UTR and 5′UTR regions (see Figure 2). The results show that all regions possess a general non-random characteristic. The mean values range from 0.20 to 0.36, with the lowest value being for ‘Transcript’ and the highest for 5′UTR. Both the 5′UTR and 3′UTR stand at less ‘random’ than the other regions, two regions which are or similar type. Enhancers and Promoter regions are very similar, with almost the same value. These are both non-coding DNA responsible for gene regulation, and contain TFBS’s (transcription factor binding sites). This means that the regions/sequences can be grouped by functionality. 

The distance from randomness values were calculated across all dinucleotides, relative to an equivalent randomized sequence of the same composition. An average value result is in the region of 0.2, meaning that, given the mononucleotide composition of the sequences, the overall dinucleotide content is enhanced or suppressed at the level of 20%, i.e., beyond the random expectation. The result of 0.36 means that enhancement/suppression is 36% above random expectation. The level observed depends on sequence and functionality type. 

For all of the sequences analysed, equivalent shuffled and, therefore, randomised sequences were generated, and the dinucleotide odds ratios and relative abundance profiles were calculated. Paired *t*-tests for the real verses shuffled datasets showed that each of the tested genomic regions was significantly different at the 1% level to its equivalent shuffled dataset (see Supplementary Materials). This again demonstrates that all genomic regions, whether protein coding or non-protein coding, are significantly different to their random equivalents, and, therefore, possess non-random signatures and patterns. 

The distance from randomness result shows that each genomic location has a distinct distance from randomness value. The different functional locations each display a distinct signature. We also observe that some non-coding DNA is less ‘random’ than protein-coding sequence. The UTR’s are least random in this regard, and these are not protein-coding. All, though, are significantly different to the random model.

### 3.2. Distance from Randomness: Binary Components RY/WS/KM 

**Figure 3:**
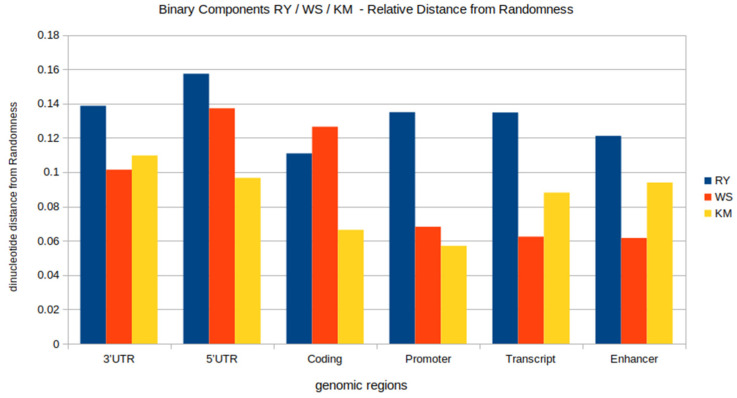
Distance from randomness for the different genomic regions, using binary components analysis (RY/WS/KM) of sequences. The profile shows the average (mean) distance from randomness of the different genomic regions, including; the 5′UTR, 3′UTR, protein-coding, promoter, transcript, and enhancer sequences. The relative dinucleotide distance from randomness for each of the RY, WS, and KM sequences for the genomic regions is shown. These are distinct sequence regions, each with their own type of functionality, and each one displays a distinct profile which distinguishes it from the other genomic sequence locations. The RY binary component is least random of most of the genomic regions, including 3′UTR, 5′UTR, the promoter, transcript and enhancer. All of these are non-protein coding sequences, with the exception of transcript. Whilst the transcript contains protein-coding sequences, it is mostly non-coding. The WS binary component is least random only of the coding DNA. In most other locations, this is the most random component. The KM binary component varies depending on sequence type; however, it is never the least random feature in any of the genomic regions analysed. A marked distinction, though, is seen between promoters and enhancers. In the promoter, it is the most random component, whereas in the enhancer it is not. This marks a potential distinguishing feature between these two types of non-coding DNA. This analysis permits a within-region comparison of relative RY, WS, and KM non-randomness.

When we view the DNA as binary-converted RY, WS, and KM sequences, and cross compare these for dinucleotide relative abundance profiles and relative distance from randomness, further layers of information are revealed (see Figure 3). The binary components and the relative distance from randomness have a distinct profile in each individual genomic region. Each is unique with respect to relative RY, WS, and KM non-randomness. This likely reflects sequence functionality, and although each has a distinct profile, there are certain groupings observed here. The different use and importance of each of the binary components RY/WS/KM is demonstrated by their distinct signatures at each location.

The results show that for each individual genomic locations the sequences are non-random in character. This is true for each of the binary components. For each of the RY, WS, and KM components, randomised-shuffled sequences were generated, which are equivalent to the actual real sequences. The results of the paired *t*-tests revealed that the sequences were significantly different (at the 1% level of significance) to the randomised ones (see Supplementary Materials). This is true for each of the binary components, and also in all of the genomic locations. Therefore all are non-random. 

The signature and relative randomness of each RY, WS, and KM binary component was also observed and compared within each individual genomic region. Each of the three binary components displays a distinct signature within a given genomic locations. The within-subject ANOVA’s for comparison of RY, WS, and KM data within a given genomic location dataset showed that these were significantly different at the 1% level of significance. This result was true for and within each of the individual genomic locations, and the same was true for coding, transcript, promoter, enhancer, 5′UTR, and 3′UTR datasets (see Supplementary Materials). The results indicate that for all 6 sequence datasets there is a highly significant effect of analysis type, the effect size being large in 5 out of 6 sequences and medium in the 3-UTR instance. To further explore the differences between analysis types, ANOVA’s were followed up in the form of post-hoc multiple pairwise comparisons corrected by the Bonferroni method. Once again, all test results were found to be highly statistically significant on all comparisons (Supplementary Materials—Tables SX and SY.). 

In a cross comparison of the profiles between the genomic locations and sequence types, we see distinct differences in coding sequence in comparison to all other locations. Whilst non-coding sequences share a general type of feature or code, these also possess some distinguishing patterns. 

The breakdown of these profiles into binary components reveals that each genomic location possesses its own unique signature with respect to RY/KM/WS and that these serve to further differentiate (beyond the ATCG relative abundance profile) each genomic location. It also reveals that information content is distinct in the protein-coding sequence compared to all other locations. For the RY/KM/WS relative abundance profiles, for instance, promoter DNA contains a very different profile to coding DNA. This reflects profoundly different information content and function. There are also differences between the promoter and other non-coding regions; however, these are less profound.

The importance of information content in coding and also transcript DNA is: WS, followed RY, then KM. Coding sequence has distance from randomness values of RY: 0.13, WS: 0.11, and KM: 0.06, and, therefore, WS is least random, followed by RY and then KM (which is most random in the sequence). It is only in the coding DNA that WS is the least random (comparatively) of the three binary components, which indicates that WS represents the most important information content in protein-coding regions. All non-coding DNA analysed here have RY as the least random of all three binary components. Therefore, in non-coding DNA in general, RY information content may be considered the most important of the three. 

Transcript values for the binary component relative abundance profiles are similar to the promoter with respect to relative non-randomness, with RY being the least random at 0.13, followed by KM: 0.0.8, and then WS: 0.06. The transcript sequence is very different to coding even though it contains coding DNA. In fact, its profile is more similar to non-coding DNA, with RY being least random. Since transcript is a mixture of coding and non-coding DNA, the general similarity, though, is likely due to the large proportion of non-coding DNA in the transcript. 

The promoter has values of RY (least random) at 0.13, followed by WS: 0.06, and then KM: 0.05, which are more random. This implies increased RY importance at promoter, and decreased WS and KM importance. The RY result suggests a prominent RY signature/code in the promoter sequence. This is also in-line with genomic DNA, and non-coding DNA in general. 

The enhancer also displays RY as the least random. The values are RY: 0.12, followed by KM: 0.09, and then WS: 0.06, which is most random. Promoters and enhancers, in terms of functionality, have some overlap. Both are non-coding, and contain an abundance of TFBS’s. Sequence features that distinguish these two regions are, therefore, worth noting. Whilst RY is least random in both the promoter and enhancer, the KM and WS profiles are different in relative randomness order. In the promoter KM is least random, and in the enhancer WS is least random, and so these potentially mark sequence properties that make these regions different. 

The 3′UTR and 5′UTR each possess a unique binary components profile, which are very different to one another. The 3′UTR has the following relative abundance profile values: RY = 0.14, KM = 0.11, and WS = 0.10, and so RY is least random, followed by KM, and WS being most random. The 5′UTR has the following values: RY: 0.16, WS: 0.14, and KM: 0.10, and so RY is least random, followed by WS, and then KM. Both of the UTR regions have relatively (similar) high and non-random RY values, in line with non-coding DNA in general. However, the non-random effect in the UTR’s is higher than any other non-coding region studied here. The two UTR’s, though, have distinct and separate binary profiles. The order of distance from randomness is inverted with respect to WS and KM. This is a strong distinguishing feature, which relates directly to sequence. In the 5′UTR, WS is most random, whereas in the 3′UTR, KM is most random. 

One pattern common to almost all genomic locations is the RY binary component, which has the highest relative abundance values (least random) compared to both KM and WS. The protein-coding sequence is the only exception, where WS values were higher. For the WS binary component, the results were more varied. In the case of the 3′UTR, enhancer and transcript sequences, the lowest relative abundance levels were for the WS binary component. In comparison, within 5′UTR and coding sequences, WS levels were more distant from randomness compared to the KM binary component. The statistics also showed that in the case of the promoter, while the WS measurements were still found to be higher than KM ones, the difference was substantially less compared to other sequence types.

In summary, considering the results for each of the binary components, RY is least random for all non-coding regions studied. This is a feature in all the non-coding, and genomic, DNA in general. WS is least random only for protein-coding sequence, and this is an unusual feature which distinguishes only coding DNA. The KM property is never the least-random feature. Either KM / WS are the most random in all regions. Information content, though, is seen to be distinct in the different sequence types.

### 3.3. Odds Ratios and Genomic Signatures: Original ATCG Sequence 

**Figure 4:**
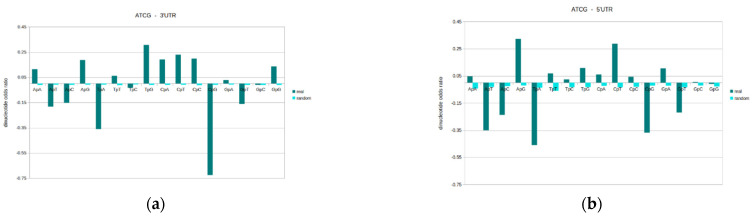

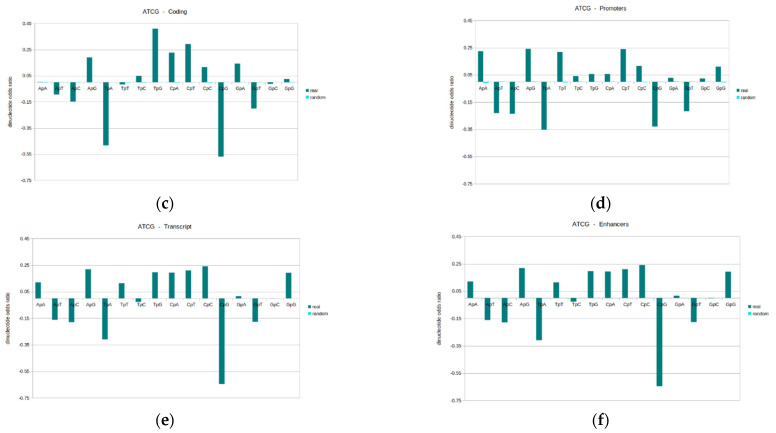
General designs profiles for the original ATCG sequence, according to genomic location These charts show the dinucleotide odds ratio profiles for the original ATCG sequence, in each of genomic sequence locations. These locations include the (**a**) 3′UTR, (**b**) 5′UTR, (**c**) coding, (**d**) promoter, (**e**) transcript, and (**f**) enhancer profiles. For each of these, the dinucleotides odds ratios are calculated for all possible 16 dinucleotides, and results here are the mean values (then standardized to 1) within the given genomic sequence type. In addition to the actual genomic DNA sequences, equivalent values for random-shuffled sequences are shown. A value of zero denotes the theoretical random model, and the further a value is from zero, the more distant that dinucleotide is from the random model. Each genomic sequence location possess a unique general design profile, which distinguishes it from all other sequence types. The most under-represented dinucleotide in all the sequence types is CpG followed by TpA. This is in-line with the general observation from genomic chromosomal DNA. The one exception is the promoter, where TpA is more strongly under-represented than CpG. This marks an important distinguishing feature of the promoter. Other under-represented dinucleotides include: ApT, ApC, GpT. The following dinucleotides are generally over-represented in the various sequence types: ApG, TpG, CpA, CpT, CpC, GpG. Additional odds ratio values that distinguish the promoter from the other genomic locations are that ApA, GpC, GpG, whilst not random, are close to this only in promoter. Features that distinguish between the promoter and enhancer are TpG, CpA, and CpC, which are more over-represented in the enhancer, and CpG, which is more suppressed in the enhancer. These general designs are extremely valuable both for potentially identifying the different sequences, and, importantly, for understanding their differences and how these relate to their particular functions in the genome.

General design profiles were generated for the original ATCG DNA sequence, in each of genomic sequence locations. The dinucleotides odds ratio results are given for all possible sixteen dinucleotides (ApA, ApT, ApC, ApG, TpA, TpT, TpC, TpG, CpA, CpT, CpC, CpG, GpA, GpT, GpC, GpG), and results here are the mean values within the given genomic sequence type (see Figure 4). Please also see Supplementary Materials 1 for full descriptive statistics. In addition to the actual genomic DNA sequences, equivalent odds ratio values for random-shuffled sequences are used as a comparison. An odds ratio value of zero denotes the theoretical random model, and the further a value is from zero, the more distant that dinucleotide is from the random model. 

The results reveal that for odds ratios and overall general designs, each genomic location has a unique profile. Whilst there is a wide-spread tendency for specific dinucleotides to be either over-/under-represented, the different types of DNA sequence possess variations in odds ratios. The complete profile consisting of all the 16 possible dinucleotide are distinct in each sequence type. The structural and functional differences of the different genomic regions are likely reflected in varying dinucleotide properties. Therefore, whilst there are general common trends, each genomic sequence location possesses a unique general design profile, which distinguishes regions. 

An odds ratio (standardised to 1) value of zero is the random expectation, or expected value for a randomised sequence of equivalent mononucleotide composition. Values above zero mean that the dinucleotide is over-represented (above the random expectation), and a negative value means that it is under-represented in the sequences. In general, the results reveal that dinucleotides that are under-represented are under-represented in all the different genomic regions to a greater or lesser degree. The same is true regarding over-representation. However, there are some notable exceptions.

The most under-represented dinucleotide in all the genomic locations examined is CpG followed by TpA. CpG is the most suppressed and is by far the most distant from randomness. The relatively low abundance of CpG and TpA fits well with their general under-representation in human genomic DNA in human chromosomes, and also in most genomes [15]. Other under-represented dinucleotides in the genomic locations examined here include: ApT, ApC, GpT. The following dinucleotides are generally over-represented in the various sequence types: ApG, TpG, CpA, CpT, CpC, GpG. 

The results of this experiment show one exception to the general CpG ‘rule’ of being the most suppressed dinucleotide. This is within the promoter, where TpA is more strongly under-represented than CpG. This goes against the grain of what is commonly seen in DNA. This is important, as it marks a distinguishing feature. Additional features that distinguish the promoter are that ApA, GpC, GpG, whilst not random, are closer to randomness than other locations. 

Additionally, features that distinguish between the promoter and enhancer are TpG, CpA, and CpC, which are more over-represented in the enhancer, and CpG, which is more suppressed in the enhancer. Since both these genomic sequences contain regulatory elements, they are differentiating features of value. The results highlight that general designs are extremely valuable both for potentially identifying the sequence type, and for understanding their differences and how these relate to their particular functions. 

### 3.4. Odds Ratios and Genomic Signatures: RY/WS/KM Binary Components 

**Figure 5:**
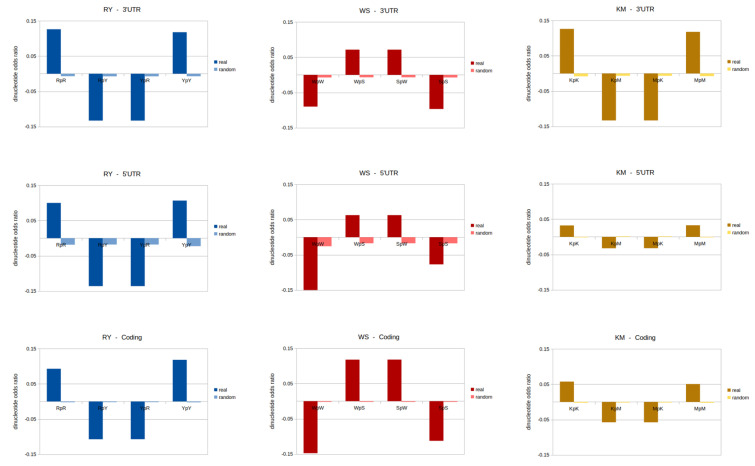

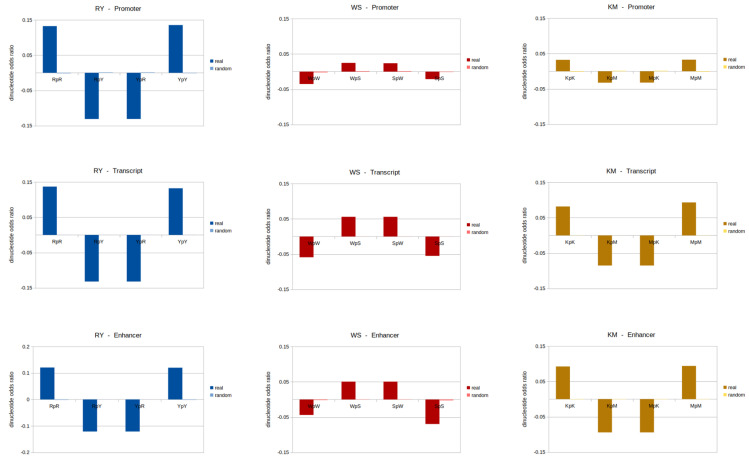
General design profiles for the RY, WS, and KM binary components, according to genomic location. These charts show the general designs profile for each binary component RY, WS, and KM separately, in each of genomic sequence locations. These locations include the 3′UTR, 5′UTR, coding, promoter, transcript, and enhancer. For each binary components, the dinucleotides odds ratios are calculated for all possible four dinucleotides, and results here are the mean values within the given genomic sequence type. Equivalent values for random-shuffled sequences are shown. A value of zero is equivalent to the random model.

The results show the general design profile for each binary component RY, WS, and KM separately, in each of genomic sequence locations (see Figure 5). Please also see Supplementary Materials 2 for full descriptive statistics. For each binary component, the dinucleotides odds ratios were calculated for all possible four dinucleotides, and results here are the mean values within the genomic sequence type. In addition to the real genomic DNA sequences, equivalent values for the random model are presented. General trends are observed in the data as well as some marked distinctions between genomic locations. 

RY: The dinucleotides RpR and YpY are relatively strongly over-represented in most locations, and RpY and YpR are under-represented throughout. These values are lowest (closer to the random model) for the coding and enhancer regions. 

WS: The dinucleotides WpS and SpW are over-represented, and SpS and WpW are under-represented in all locations. However, these are much closer to the random model in the promoter. The strongest levels of enhancement/suppression are in the coding region for all the WS dinucleotides. 

KM: The dinucleotides KpK and MpM are over-represented, and KpM and MpK are under-represented in all locations. However, these are much closer to the random model in the promoter and 5′UTR.

These results show that each genomic location possesses its own unique genomic signature. The binary component odds ratio values are also distinct in each location. However, there is a common theme for each of the RY, WS, and KM binary components. Over-represented dinucleotides are this way in all regions, and under-represented are so throughout. This means that the binary component odds ratios display coherence. The difference between genomic locations is only with respect to the extent of enhancement or suppression. The random-shuffled equivalent sequences have odds ratio values close to zero in all the locations. These are slightly higher in the UTR, which is likely due to these being shorter sequence segments. In longer segments, the random values are closer the theoretical/true random value of zero.

Within the RY binary component dataset, the dinucleotides RpR and YpY are strongly over-represented in all the locations, and RpY and YpR are under-represented throughout. These values are lowest (closer to the random model) for all the coding and enhancer regions. This profile is consistent for all the six genomic location datasets. However, within the coding and enhancer datasets, the odds ratios are closer to the random model than the other sequence types. The DNA generally favours homogenous dinucleotides for this binary component, and suppresses the heterogeneous ones. 

For the WS binary component, WpS and SpW are over-represented, and SpS and WpW are under-represented in all locations. However, these are much closer to the random model in the promoter. The strongest levels of enhancement/suppression are in the coding region for all the WS dinucleotides. In general for this binary component, the DNA enhances heterogeneous dinucleotides, and suppresses homogenous ones. For the KM binary component, KpK and MpM are over-represented, and KpM and MpK are under-represented in all locations. However, these are much closer to the random model in the promoter and 5′UTR. For each of the profiles, a randomised model dataset was also analysed. The results here were close to zero, as expected. For both UTRs, however, the odds ratio values were slightly higher, closer to zero, due to shorter sequence stretches of the UTRs. 

These results demonstrate that each location possesses a unique signature for each binary component. The odds ratio values are distinct in each location. However, there is a common theme for each of the RY, WS, and KM sequence sets. Over-represented dinucleotides are this way in all genomic regions, and under-represented are also this way throughout. This means that the binary component odds ratios display coherence, that DNA retains these features, and this general ‘rule’ is not disobeyed. The boundary of over and under representation is not crossed. The difference between genomic locations is only with respect to extent of dinucleotide enhancement or suppression. 

## 4. Discussion and Conclusions

### 4.1. General Designs of the ATCG Original Sequence in Different Genomic Locations 

Genomic signatures are a powerful tool and method for the analysis of sequence, and distinguishing sequence types. The genomic signature is seen to be distinct for each of the genomic locations studied. This is in-line with past experiments [21] where the dinucleotide genomic signature was shown to be pervasive, and capable of distinguishing genomic DNA of different species and also the chromosomes within a given species. This is due to the unique nature of the dinucleotide relative abundance profile. It generates a quantitative profile that is the most basic description of a sequence. In addition, it describes the random/non-random characteristics of the sequence. Furthermore, dinucleotides and the relative abundance profile reflect patterns and potential codes inherent in sequences. This makes the dinucleotide relative abundance profile a powerful tool for analysing the large-scale differences between genomic sequences. 

CpG is the most suppressed and is by far the most distant from randomness of all the dinucleotides. TpA is also under-represented. This is true in all the genomic locations studied. The CpG and TpA suppression fits well with their general under-representation in the human and many other genomes [15]. 

The general tendencies of dinucleotides to be over- or under-represented can be due to the sequence assembly tendencies of DNA, which generate a viable stable molecule. Mutation events may also generate bias. For instance, in genomic DNA in general, CpG is present at a lower level and is much more suppressed than GpC, which is not suppressed. Under-representation of CpG is thought to be due to methylation and deamination, which causes the mutation of CpG to either TpG or CpA. TpG and CpA occur at a higher than random expectation (whilst CpG occurs at a lower than expected frequency). 

One exception is the promoter, where TpA is slightly more suppressed than CpG, which is unusual in DNA. One possible explanation for this very strong and unusual under-representation of TpA is its presence as a part of regulatory motifs such as the TATA box. Since the TATA box is a fundamental element, there would be a selective pressure within DNA to under-represent this. This would help to prevent the inappropriate binding of regulatory proteins to the DNA, to possess their own characteristic sequence motifs related to their particular function. In addition, the stacking energy of TpA is the lowest of all dinucleotides, permitting the DNA to unwind. Suppressing it makes sense in this regard, as easy unwinding is not generally desired, unless for a specific reason. Also suppression helps to prevent the inappropriate binding of regulatory proteins to the DNA, or to possess their own characteristic sequence motifs related to their particular function.

The dinucleotides odds ratios and relative abundance profiles were generated for genomic sequences not previously analysed. This includes the promoter, enhancer, 5′UTR, and 3′UTR. The results show distinctions between different types of non-coding DNA, as well as the coding sequences.. There are a variety of constraints on different sequence types related to functionality and also evolutionary pressures. Profile similarities occur where overall constraints on DNA remain the same regardless of sequence type.

### 4.2. General Designs for Genomic Locations: The RY/WS/KM Binary Components 

Within the category of non-coding DNA, the promoter contains its own unique signature for the binary components, and can be distinguished from the enhancer; thus, whilst these locations share common features for gene regulation, there are also some distinct features. Within the promoter, the genomic signature for the RY sequence was most distant from the random model. This was followed by the WS and KM sequence signatures, with KM being the most random. The result implies RY codes or patterns are the most prominent or of greatest importance. Since purines/pyrimidines are determinants of structure, this suggests a potential R/Y structural code in the promoter. The WS/KM properties are determinants of hydrogen-bonding donor–acceptor patterns. The results indicate this property is less important in the promoter than the RY property of structure. WS is relatively less random than KM in the promoter. 

In the enhancer, RY is the most distant from randomness, and we conclude the same type of RY-structure-based code. This is followed by KM, and then WS, which is the most random. The KM and WS relative distance from randomness mark a distinction between the promoter and enhancer. This may be due to a difference in functionality, or it could be due the proximity of the promoter to the UTR and protein-coding region. In the enhancer, the most random property is the WS property, which relates to the hydrogen-bonding potential between base pairs. In the enhancer, this property is not as necessary. 

In addition, the odds ratio values show that all under-represented dinucleotides in the promoter and enhancer are composed of a purine and a pyrimidine, whilst most of those that are over-represented are composed of either two purines or two pyrimidines. We see that this pattern is also true in all the other genomic locations studied, it is just the extent which varies.

In the coding region, the WS component is most distant from randomness, followed by RY and then KM. The WS component is connected with the chemical property of hydrogen bonding between the base pairs of DNA, but also the specificity of tRNA binding and codon–anti-codon interactions. In protein-coding sequences, the WS property is least random due to this being inherent in the genetic code. Genomic signatures capture this. RY is less important because the structural property is least relevant here. It is not needed in this way, as the encoding is simply different. KM is the most random in the coding sequence, as the keto-amino property and major-groove donor–acceptor patterns are not required here, as in other locations. 

In contrast, in the transcript, RY is the least random followed by KM, and then WS. Even though transcript contains protein-coding DNA, the majority of sequence is intronic non-coding. The profile contrast to coding sequence reflects this. It is also worth noting that transcript has a similar distance from randomness profile to the enhancer. This also reflects the presence of enhancer and regulatory sequences within the transcript. 

The UTR’s possess a strong RY-structural code, which is much more prominent than KM and WS, reflecting the fact that structure is the predominant feature. The KM signature, though, is very different for the 5′UTR, where it is much closer to randomness, and 3′UTR, where it is relatively non-random. This suggests a different type of H-bonding activity. Since the KM signature is a determinant of major-groove hydrogen bonding, there is a likely difference in this respect between the UTR’s. 

In the RY binary component sequence dataset, there are four possible dinucleotides, which are RpY, RpR, YpR, and YpY. The dinucleotides YpY/RpR are enhanced above the random expectation given the nucleotide content, and RpY/YpR are suppressed. The RpY/YpR dinucleotides are flexible at the local level of secondary DNA structure, whilst RpR/YpY are rigid, and so we can conclude that, in general, in all the locations investigated, flexibility is suppressed whilst rigidity is enhanced. All in all, the RY property is a structural one. This result is also supported by research with Erdös motifs, showing that certain RY motifs of 10 bp length are under-represented in the human genome [40]. 

This general design profile is consistent for all six genomic locations studied, which reflects a general tendency in genomic DNA. There is coherence here, and this baseline ‘rule’ is adhered to throughout. It likely related to the stability of DNA, and the way in which it, by nature, assembles. The difference between genomic locations is in the level of the suppression/enhancement of these RY dinucleotides. RY forms a strong feature in all non-coding DNA. In contrast, coding DNA has a very different nature. 

For the KM binary component: KpM / MpK are suppressed, whilst KpK / MpM are enhanced all the genomic locations analysed. However, in the promoter and 5′UTR they are much closer to randomness than all other locations. In addition, the extent of suppression/enhancement varies greatly between the genomic locations. This property and pattern directly affects hydrogen-bonding donor/acceptor sites, and distinguishes these at the major groove of DNA. These binding patterns are related to the recognition and specificity of binding to biological particles. The results show that either sequential donors or sequential acceptors are favoured. This may be a pattern favourable for the binding of particles to DNA. 

In the WS binary component dataset, WpW and SpS are suppressed, whilst WpS and SpW are enhanced, and this is true across all the genomic regions analysed. The only exception is the promoter, in which the level of enhancement/suppression, whilst not random, is closest to it, of all locations. DNA in the different sequence types generally favours and enhances dinucleotides with a heterogeneous hydrogen-bonding donor/acceptor pattern in the minor groove, and also a heterogeneous number of hydrogen bonds between the base pairs. In contrast, the same or homogeneous patterns are suppressed. This heterogeneous pattern of hydrogen bonding between base pairs may be favoured, as it increases the stability of local secondary helical structures. For coding sequences, WS is the least random component. This reveals that within the triplet code, the hydrogen bonding between the base-pairs is the most important property.. In other words, it functions through complementary base pairing. We see that the components analysis captures this encoding, and the chemical boding property, namely, hydrogen bonds. In contrast, within the promoter, these patterns are closer to randomness, as they are not needed nor as important in this location. 

### 4.3. Binary Components Reveal Distinct Patterns/Codes in Coding and Non-Coding DNA 

In our previous research [33], we established proof of principle for binary components analysis with general designs. Here, we looked at large-scale regions of chromosomal DNA, and discovered the presence of an RY structural code across all human chromosomes. In this present research, we analyse distinct functional genomic regions for comparison, namely, coding sequence, transcript, promoter, enhancer, the 5′UTR and 3′UTR. We investigate whether the general designs are different, and seek to understand their encoding. 

The binary components of the four bases reflect and separate out the chemical and physical properties of DNA. This analysis leads to insight into patterns and codes. The RY component is a physical property that determines secondary structure [32]. In contrast to this, the KM and WS components determine the chemical property of the bases, including hydrogen-bonding capability (and patterns). It is known that the RY make up of DNA determines the relative flexibility/rigidity of the molecule, and ability to bend [41,42]. At any given segment of the DNA, this will change depending on the particular stretch of sequence. The analysis of the non-randomness of identical sequences with respect to each of these three binary components here separates these properties, and then determines their relative importance in the different sequences. This permits the comparison of protein-coding and non-coding DNA. 

With respect to binary components profiles, the non-coding genomic regions are generally more similar to each other than to the coding DNA. This is to be expected, since protein-coding regions are very distinct in their task of coding for peptides. In general, non-coding regions are much more non-random in their RY signatures, and so structural patterns define non-coding DNA. In contrast, the coding sequences examined were much less random in their WS/KM signatures than the RY signature. The binary-components analysis permit us to delve into these distinct sequence features, which are due to a different type of encoding. 

The protein-coding DNA displayed the WS binary component as its most prominent (least-random) feature. This reveals a general WS chemical code in the coding DNA. The triplet code actually depends on hydrogen-bonding binding, since it specifies codon–anti-codon binding. It depends on differentiating between hydrogen bonds between tRNA and the DNA. The WS property defines this. The general designs with binary components analysis actually captures this encoding. 

All the types of non-coding DNA have the RY component as their most prominent (least-random) feature, suggesting an RY-structural-based code in all non-coding regions. This observation falls in line with the fact that much of the non-coding DNA function entails binding to protein particles. This binding requires recognition specificity, which may be largely structural in nature. The RY property is, therefore, critical to this functioning of non-coding DNA. None of the genomic locations analysed has the KM (keto/Amino) property as the most prominent feature; it is either the most random or second to most random component. 

The binary components analysis adds a powerful dimension to the analysis, by breaking down information content. Therefore, it is employed here to investigate differences in underlying patterns and codes between genomic regions. Breaking down the DNA nucleic acids sequence into component parts (RY/WS/KM) according to chemical and physical properties allows us to see these properties in isolation. It permits these properties of general designs to be compared in the coding and non-coding DNA. 

### 4.4. The Connection between Functionality and Codes

There exists a sequence–structure–function connection, and, therefore, the sequence patterns inherent in the non-coding DNA are expected to be different to protein-coding DNA. The genomic signature results seen here reflect this. This signature difference shows that different characteristic words or patterns that exist within the DNA sequence are inherent to the different genomic regions. This, in turn, implies distinct codes. The distinct functionality relates directly to DNA properties, and also sequence patterns and underlying codes. 

The promoter is responsible for transcription regulation, and possesses the necessary DNA patterns or potential ‘codes‘ for this task. Transcription regulation is governed by a biological machinery which interacts with DNA at specific locations. The basal machinery binds around the TSS, and the promoter region contains this, as well as other TFBS’s that regulate transcription. The enhancer is also dense in TFBS’s. The information content of the DNA and its sequence is critically important for this process. This is because of the inherent interrelationship between DNA sequence, structure (both secondary and tertiary), and function. The regulatory function of this DNA makes it unique and its signatures distinct from other genomic locations (with different functions). 

The results strongly suggest an RY code which is structural in nature, in the promoter and enhancer regions. A KM/WS chemical code is also present but of lesser importance. Given existing knowledge of the biological function of the promoter and enhancer, this makes sense. The promoter is extremely dense in protein binding sites, and for transcription regulation, it is necessary for TF’s/proteins to specifically recognise their binding sites. These are short stretches of DNA (6–20 bp’s in length). Presently, we do know how this specificity is attained, and what constitutes the code(s) for this. Protein–DNA binding requires structural fit or compatibility between the DNA and protein, and is, therefore, highly structural in nature. This explains the connection with a structural RY code. Non-coding DNA may, in general, require more structural specificity than protein-coding DNA. Whilst we know that there is not a direct rule-driven code in non-coding DNA, the results do point towards complex patterns and codes. Whilst these are not simple, they are present and require examination. 

In contrast, coding DNA is very different in nature. The most prominent feature being the triplet code. The structural motifs of the DNA sequence is less important here, in terms of secondary and tertiary structure. Rather, the chemical properties of hydrogen-bonding are more significant, which explains the relative importance of the WS signature.

### 4.5. Assumptions and Limitations 

There are some limitations with the sequence datasets. These include the predictive nature of the sequence type and boundaries of sequence locations. This limitation has been minimised as much as possible by utilising datasets that have been well curated and based on experimental evidence. Nevertheless, there are imperfections, particularly with boundaries, i.e., where a sequence type begins and ends. For the promoter and 5′UTR, the precise location of the TSS (transcription start site), may be inaccurate. The VISTA enhancer dataset used here was specifically chosen because all these are experimentally elucidated and not based only on prediction. However, the boundaries here are likely larger than the true enhancer sequence. This inaccuracy may slightly alter the outcome. The various sequence types have such inaccuracies that could affect the results, and any subsequent sequence analysis. 

Another issue or limitation is to do with length of sequences. Inherently, the different sequence types vary, and can be short. In order to study regions such as coding and promoters, etc., you must deal with short sequences. Dinucleotide odds ratios and relative abundance profiles are easier to analyse with longer sequences, as the results more accurately approach the random model. Here, we adapt to analyse shorter stretches. To eliminate noise we utilise very large datasets, and also generate an equivalent randomised model to ensure accuracy of results.

Both the odds ratio values and the connected dinucleotide relative abundance profile are a measure of the average over- or under-representation of dinucleotides in a given sequence. The distance from randomness measure is adapted to assess the overall average randomness (or non-randomness) of a sequence in comparison to the theoretical random one. The binary components analysis for the RY, KM, WS binarized sequences takes this a step further. Here, we compare the relative randomness (or non-randomness) for each of the RY, KM, and WS properties for the exact same sequences. The assumption (and interpretation), though, that we make here is that relative non-randomness is analogous with greater importance of that component. We interpret this as the component having patterns in a sequence. Whilst this is a reasonable conclusion, we must state that this is an assumption. 

It is then possible to compare the general design profiles of the coding sequences, promoter, enhancer, etc., and correlate these with the function of those sequences. Sequence and function are inherently linked. This means that it becomes possible to draw conclusions with the binary component results with function of the sequences. There are some assumptions. We assume that there is a connection of the RY, WS and KM component with physical/chemical properties. This includes that the RY property of a sequence is connected with its structure. This correlated with existing evidence for DNA structure. A further assumption is that WS and KM are linked with the hydrogen bonding capacity. This too makes sense and there is strong evidence in this direction. However, the coherence is not necessarily precise. We say this because both WS and KM have other properties, and not solely hydrogen-bonding capacity. 

### 4.6. Further Research 

The binary components with general designs can be used to distinguish genomic regions, and can be applied to their identification. The outcome leads to two main paths of further research. The first is to better understand sequence patterns and codes within the promoter and other non-coding DNA. This enables a better understanding of the DNA and its role in transcription and gene regulation in general. The second path is the use of the genomic signatures (with binary components) to identify and predict genomic locations. There are other methods that may be developed for further analysis of patterns in the promoter and other genomic sequences. 

The RY structural code, particularly in the promoter (but also other locations), can be further investigated. This would involve analysis of sequence patterns in the promoter. Known TFBS’s and their DNA sequences may also be examined further for patterns and related structural features. On a wider scope, different genomic locations may be studied for DNA signatures, and a full map produced for other non-coding locations. The signatures analysis may of course be expanded to other organisms. We strongly suspect general principles of encoding will be similar, since these are likely common features of non-coding DNA across genomes. In addition, structural features of the DNA can be studied and correlated with these observations.

The second path of investigation is the prediction of genome architecture, coding/non-coding, promoter and TSS. The results of this paper may be used to generate learning algorithms that utilize genomic signatures with binary components to improve predictions for differentiating coding and non-coding DNA. Further research would include the development of algorithms that ‘reverse engineer’ these findings to accurately identify and predict sequence type. 

### 4.7. Concluding Remarks 

The purpose of this investigation is to decipher patterns and codes in different types of genomic DNA. These findings bring about two very significant outcomes: The first is for building a profile of sequence patterns and signatures for the different sequence types. This permits a deciphering of underlying codes, which are related to distinct functions of different regions of DNA. This allows us to also understand the connection between sequence, structure and function, which is fundamental to biology. The second is creating a profile that distinguishes between the different genomic sequences, and this profile could be utilised in a predictive sense. 

With the binary comportments analysis, we used the principal of breaking down the information stored within the DNA base sequence to better understand function and decipher codes. This is much like the use of computer binary code for the transmission of complex information. This information can be layered or multidimensional, yet transmitted in binary code.

In summary, the findings show that there is a difference in the general designs between protein-coding DNA and non-coding DNA. The RY, WS, and KM binary components signatures distinguish between these. We conclude from the findings that in protein-coding DNA there is a primarily WS-chemical-based code, whereas in non-coding DNA there is a dominant RY-structural-based code. This is a novel and impactful result. 

Furthermore, this dominant RY code is observed in the different types of non-coding DNA. We also conclude that these codes are connected to function. In the non-coding DNA such as the promoter, an RY-structural code is dominant, and hydrogen-bonding less important, which likely reflects the role of this DNA in transcription regulation. In contrast, coding DNA has a completely different binary components nature, due to the hydrogen-bonding requirement for codon–anti-codon interaction. This research marks a step forward towards understanding the encoding of functionally distinct locations of DNA. 

This research makes use of the inherent relationship between sequence, structure and function, and, therefore, biological meaning is successfully derived from sequence data. It aids the deciphering of codes. These findings have implications for understanding how non-coding DNA functions and for gene regulation. It helps us to determine the difference between the genetic code and other codes. These findings have implications for the most fundamental principles of biology. Furthermore, this can assist in understanding of cellular growth and development and in knowledge of disease states. 

## Data Availability

Data is contained within the article or supplementary material.

## Supplementary Materials

The following supporting information can be downloaded at: https://www.mdpi.com/article/10.3390/genes13111970/s1, file S1: suppl_descriptive_statistics_original-ATCG.ods; file S2: suppl_descriptive_statistics_binary-RY-WS-KM.ods; file S3: A ‘nearest neighbour’ step or dinucleotide in DNA in the 5′ to 3′ orientation; file S4: ANOVA results table for distance from randomness of binary components is found in the following attached file: appendix_ANOVA_binary-RY-WS-KM.ods.; file S5: Paired t-test results for real verses random model data is found in the following attached file: appendix_paired_Ttest_real_vs_random.ods.

## Appendix A

https://proteomesci.biomedcentral.com/articles/10.1186/1477-5956-2-3. Copyright © 2004 Höglund and Kohlbacher; licensee BioMed Central Ltd. This is an Open Access article: verbatim copying and redistribution of this article are permitted in all media for any purpose, provided this notice is preserved along with the article’s original URL. https://www.biomedcentral.com/about/policies/license-agreement. https://creativecommons.org/licenses/by/4.0/.
