# Supplementary material for: General Designs Reveal Distinct Codes in Protein-Coding and Non-Coding Human DNA
Source: Genes (Basel). 2022 Oct 28;13(11):1970. doi: 10.3390/genes13111970 (PMC9690640; doi:10.3390/genes13111970)
Supplement: Supplementary file 1 [file genes-13-01970-s001.zip › suppl-fileS3_A í«nearest neighbourí» step or dinucleotide in DNA in the 5íΣ to 3íΣ orientation.pdf]

The notation ApA, ApC, ApT etc….. denotes a 'nearest neighbour' step or dinucleotide in DNA in the 5′ to 3′ orientation, along the phosphate backbone. It is given/used as a biochemical distinction between a variety of different possible doublets.
